# An enhancer RNA-based risk model for prediction of bladder cancer prognosis

**DOI:** 10.3389/fmed.2022.979542

**Published:** 2022-09-14

**Authors:** Zhicheng Xu, Chao Xu, Qionghan Wang, Shanjin Ma, Yu Li, Shaojie Liu, Shiyuan Peng, Jidong Tan, Xiaolong Zhao, Donghui Han, Keying Zhang, Lijun Yang

**Affiliations:** ^1^Department of Urology, Xijing Hospital, Fourth Military Medical University, Xi’an, China; ^2^School of Basic Medicine, Fourth Military Medical University, Xi’an, China; ^3^Department of Urology, Tangdu Hospital, Fourth Military Medical University, Xi’an, China; ^4^96607 Army Hospital of People’s Liberation Army, Baoji, China

**Keywords:** prognostic model, bladder cancer, enhancer RNA, WGCNA, tumor invasion

## Abstract

**Background:**

Bladder cancer patients have a high recurrence and poor survival rates worldwide. Early diagnosis and intervention are the cornerstones for favorable prognosis. However, commonly used predictive tools cannot meet clinical needs because of their insufficient accuracy.

**Methods:**

We have developed an enhancer RNA (eRNA)-based signature to improve the prediction for bladder cancer prognosis. First, we analyzed differentially expressed eRNAs in gene expression profiles and clinical data for bladder cancer from The Cancer Genome Atlas database. Then, we constructed a risk model for prognosis of bladder cancer patients, and analyzed the correlation between this model and tumor microenvironment (TME). Finally, regulatory network of downstream genes of eRNA in the model was constructed by WGCNA and enrichment analysis, then Real-time quantitative PCR verified the differentiation of related genes between tumor and adjacent tissue.

**Results:**

We first constructed a risk model composed of eight eRNAs, and found the risk model could be an independent risk factor to predict the prognosis of bladder cancer. Then, the log-rank test and time-dependent ROC curve analysis shown the model has a favorable ability to predict prognosis. The eight risk eRNAs may participate in disease progression by regulating cell adhesion and invasion, and up-regulating immune checkpoints to suppress the immunity in TME. mRNA level change in related genes further validated regulatory roles of eRNAs in bladder cancer. In summary, we constructed an eRNA-based risk model and confirmed that the model could predict the prognosis of bladder cancer patients.

## Introduction

Bladder cancer is the tenth most common source of malignancy with a clear predominance mortality worldwide (1). Approximately 25% of bladder cancer patients are diagnosed with muscle-invasive bladder cancer or metastatic disease (2), which contributed to 36–42% and 5% (distant) to 36% (regional) 5-years overall survival respectively (3). Although survival of patients with non-muscle-invasive bladder cancer is favorable, the cost for repeated endoscopic assessments and resections still remains expensive (4, 5). Furthermore, bladder cancer patients still experience high rates of recurrence, more than 50% within 3 years (20% recurrence within only 1 year) (6, 7). Recently, new therapeutic strategies such as immune checkpoint inhibitors have been developed (8), however, progression in bladder cancer survival is still unsatisfactory (9). Hence, with less improvement in prolonging survival of bladder cancer patients, finding biomarkers predicting the onset and progress of the disease for early screening and prognostic evaluation may provide us with a new pathway to promote the current status of diagnosis and treatment of bladder cancer.

Enhancers are *cis*-regulatory DNA elements that cooperate with promoters to increase transcription of genes (10, 11), and the abnormal status of enhancers may promote the occurrence and progression of tumors (12). Reports have shown that activated enhancers can recruit RNA transcription elements to produce a class of non-coding RNAs (ncRNAs), known as enhancer RNAs (eRNAs). Recently, increasing evidence has demonstrated the roles eRNAs played in the regulation of body physiological and pathological processes (13, 14). Mechanically, eRNAs participate in the transcription of target genes and facilitate the transcription process by driving enhancer-promoter looping, regulating the recruitment and activities of transcription factors and promoting RNA pol II pause-release (15–18). Moreover, transcription of eRNAs precedes transcription of adjacent mRNAs during the process of gene expression (19, 20), making them indicators of the expression level of target genes. Thus, the genome-wide expression and specificity of eRNAs in different tumor types suggest that eRNAs could be used for early diagnosis of asymptomatic patients, predict tumor prognosis, and serve as therapeutic targets (21–24).

In the present study, we constructed an eRNA-based risk model to predict the prognosis of bladder cancer and explore the possible downstream mechanisms which the risk eRNAs may affect. First, we conducted an integrated analysis of eRNA expression and construct a risk model based on survival-related eRNAs for predicting the prognosis of bladder cancer patients. Then, we analyzed correlations between the risk model and clinicopathological features and tumor microenvironment. Lastly, we performed weighted gene co-expression network analysis (WGCNA) and enrichment analysis to analyze eRNA-related target genes to further illustrate the regulatory mechanism.

## Materials and methods

### Data collection

Clinical information and fragments per kilobase of transcript per million fragments data for bladder cancer eRNAs were downloaded from The Cancer Genome Atlas portal,^1^ which includes 414 bladder cancer and 19 non-tumor datasets. After matching transcriptomic data and clinical information with ID numbers, patients with follow-up time < 90 d or other incomplete data were removed. Genes associated with eRNAs were predicted with the enhancer RNA in cancer (eRic) website.^2^ All data were processed and analyzed with R software.^3^ The 414 bladder cancer samples were randomly slitted into two of the validation sets.

### Cox regression analysis

We used Wilcoxon signed-rank test to analyze eRNAs differently expressed between tumor and normal tissues. *P*-value was adjusted with false discovery rate (FDR), and filter criteria were confirmed as FDR < 0.05 and | log2 fold-change (FC) > 1. Survival-associated eRNAs were identified by univariable Cox regression analysis with a threshold of *p*-value < 0.01. The overall survival curves attest to the fulfillment of the assumption of proportional hazards in the Cox regression models. *p*-value was calculated by log-rank test.

### Construction and confirmation of eRNA-based prognostic signature

An eRNA-based risk model was constructed by multivariate Cox regression. Survival R package was adopted to construct a survival eRNAs-based prognostic model. To avoid overfitting, the 19 survival -related eRNAs that correlated highly with other genes were deleted. Then, Cox proportional hazards regression was used to build a prognostic risk model, and the regression coefficients were used to weight the expression value of selected eRNAs. Following this, samples were randomly split into two of validation sets. Then Kaplan-Meier curve analysis was performed by the log-rank test, and receiver operating characteristic (ROC) analysis was used to evaluate the prediction ability of the model for the training and validation cohorts.

To improve clinical applicability, we further constructed a systematic signature by integrating clinicopathological variables with risk score generated by the aforementioned risk model and prepared a nomogram with rms R package.

### Independent prognostic factor analysis

Univariate and multivariate Cox regression analysis was used to recognize factors (i.e., commonly used clinicopathological variables and the risk score of the present model) that could independently affect the overall survival of bladder cancer patients. A *p* < 0.05 was considered to represent statistical significance.

### Clinical application of the prognostic signature and tumor microenvironment analysis

The relationship between risk factors in the present model (i.e., risk score and related eRNAs) and selected clinical variables (age, gender, histological grade, pathological stage, and TNM status) was analyzed with the Wilcoxon test. The Box plot and Kaplan-Meier survival curves were prepared with the beeswarm R package and survival R package, respectively. Subsequently, tumor-infiltrating immune cells of each bladder cancer patient were analyzed and quantified by ESTIMATE and MCPOCUNTER method (25), and the correlation between risk score and infiltrating immune cells was measured by the Pearson correlation coefficient test.

### Weighted gene co-expression network analysis algorithm and enrichment analysis

Enhancer RNA-related target genes were analyzed with WGCNA algorithm and enrichment analysis. Briefly, the gradient method was used to select the appropriate power value with an independence degree of 0.9. Subsequently, the hierarchical clustering of the topological overlap matrix was employed to construct a dendrogram and identify the co-expressed gene sets (modules). The interaction among different modules was analyzed and visualized with R software. Afterward, the module-trait relationship was analyzed to recognize the phenotype (clinical trait) highly correlated expression set. Finally, a co-expression network of the interested module was constructed with Cytoscape software. Enrichment analysis was performed to identify a mechanism of eRNA in bladder cancer progression. An FDR < 0.05 was used as the threshold.

### Patient samples

Three pairs of tumor and para-cancerous samples used for present assay were collected from the patients undergoing radical operation in our institute. The subjects were informed and gave written consent. All protocols were authorized by the Ethics Committee of The First Affiliated Hospital of Air Force Military Medical University.

### Real-time quantitative PCR analysis

TRIzol Reagent (Cat#15596018, Invitrogen, United States) was used to isolate the total RNA of samples according to the manufacturer’s protocol. The RevertAid First Strand cDNA Synthesis Kit (Cat#RR036A, TAKARA, Japan) was used to synthesize cDNA from 500 ng of total RNA. Real-time quantitative PCR (qRT-PCR) was carried out by using TB Green Premix Ex Taq II Kit (Cat#RR820A, TAKARA, Japan) on BioRad CFX96 system. Expression levels of the target genes were normalized to a housekeeping gene GAPDH. Gene expression values are stated as 2^–ΔΔ^*^t^*. The sequences of the primers are listed in Supplementary Data 1. All results were performed by Student’s *t*-test or Linear regression using GraphPad Prism 8. Statistical analysis was expressed as mean with SEs, and differences were considered significant when a value of *p* < 0.05.

## Results

### Identification of bladder cancer-specific prognostic-related eRNAs

**FIGURE 1:**
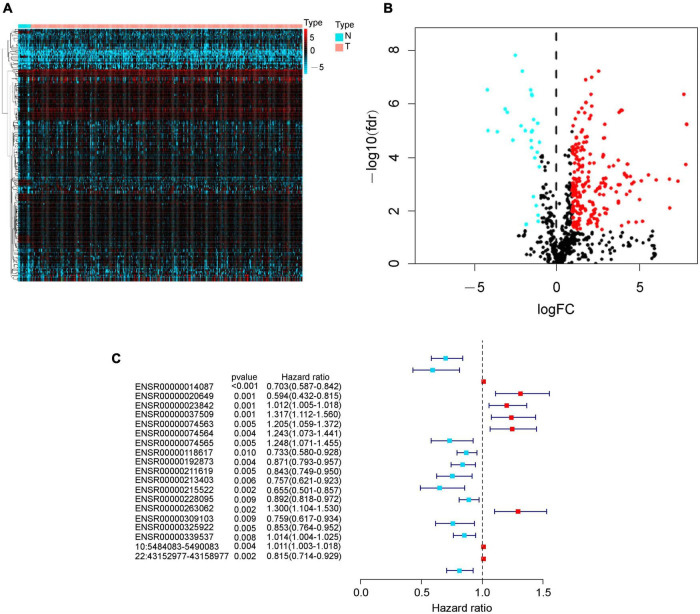
Identification of differently expressed eRNAs in bladder cancer. **(A)** Heat map of eRNAs. The blue to red spectrum represents the expression level of eRNA from low to high. **(B)** Volcano plot of eRNAs. The blue and red dots show downregulated and upregulated eRNA genes, and the black dots indicate unchanged eRNA genes. **(C)** Forest graph of survival-associated eRNAs. The red and blue dots represent eRNAs with hazard ratio > 1 and ≤ 1, respectively.

We identified 256 differently expressed eRNAs from total 832 based on The Cancer Genome Atlas bladder cancer dataset; we found 227 upregulated eRNAs and 29 downregulated eRNAs in bladder cancer tissues compared with normal tissues (Figures 1A,B). Subsequently, we identified 19 survival-associated eRNAs based on 371 eligible bladder cancer patients (Figure 1C), of which eight eRNAs were recognized as risk genes with hazard ratio > 1.

### Construction and validation of eRNAs-based prognostic risk model

After deleting eRNAs that would overfit the present model, we used multivariate Cox regression to build a prognostic risk model with eight optimal eRNAs (Table 1). Then, the risk score of each bladder cancer patient was calculated according to the following formula:

**TABLE 1:** Eight eRNAs in the risk model.

Gene	Coefficient	*P*-value	HR (95% Cl)
ENSR00000014087	−0.250412427	0.025814	0.624626495
ENSR00000020649	−0.295461512	0.086906	0.530610638
ENSR00000023842	0.01036409	0.003744	1.003362679
ENSR00000037509	4.740086045	0.012739	2.746925389
ENSR00000074564	0.251752135	0.002429	1.093099886
ENSR00000263062	−4.063099964	0.022422	0.000525543
ENSR00000309103	−0.190141695	0.095442	0.661232085
ENSR00000339537	0.021737751	0.000476	1.009588673

Risk score = (−0.25041 × expression of ENSR0000 0014087) + (−0.29546 × expression of ENSR00000020649) + (0.010364 × expression of ENSR00000023842) + (4.740086 × expression of ENSR00000037509) + (0.251752 × expression of ENSR00000074564) + (−4.0631 × expression of ENSR000 00263062) + (−0.19014 × expression of ENSR00000309103) + (0.021738 × expression of ENSR00000339537).

**FIGURE 2:**
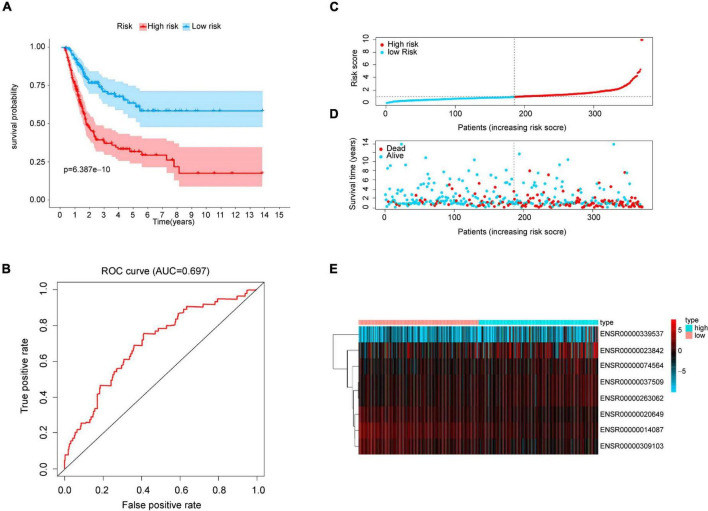
Prognostic analysis from risk model. **(A)** Kaplan-Meier curve analysis of high-risk and low-risk groups. Light-colored background around the curves indicates 95% CI. **(B)** ROC curve of the prognostic model. AUC > 0.60 was regarded as acceptable for prediction. **(C)** Risk score distribution of bladder cancer patients based on the prognostic model. **(D)** Scatter plot of survival status of two risk groups. **(E)** Heatmap of eRNAs expression.

With the median risk score based on the risk model, we sorted patients into a high-risk group and low-risk group, then Kaplan-Meier log-rank test showed that the overall survival of the high-risk group was significantly lower than that of the low-risk group in the whole cohorts (*p* < 0.001, Figure 2A) with a favorable AUC in the 5-years (AUC = 0.697, *p* < 0.05, Figure 2B), and in two of the validation sets (*p* < 0.001, Supplementary Figures 1A,C) with an AUC in 5-years OS (AUC = 0.831 and 0.658 respectively, *p* < 0.05, Supplementary Figures 1B,D). Meanwhile, OS of survival eRNAs in the model demonstrated that high expression of protective genes (i.e., ENSR00000014087, ENSR00000020649, and ENSR00000309103) was related to a favorable prognosis. In contrast, overexpressed risk genes (i.e., ENSR00000037509 and ENSR00000263062) were associated with poor prognosis (*p* < 0.05, Supplementary Figure 2), which is the same as the results Figure 1C shown. Then, a risk plot was prepared, composed of a risk score, survival status, and eRNA expression. The plot confirmed that the risk score could stratify bladder cancer patients with different survival outcomes (Figures 2C,D). Finally, the differential expression of the eight eRNAs in bladder cancer and the normal was presented by heatmap (Figure 2E).

### Independent prognostic value of the present model and clinical application of the present model

**FIGURE 3:**
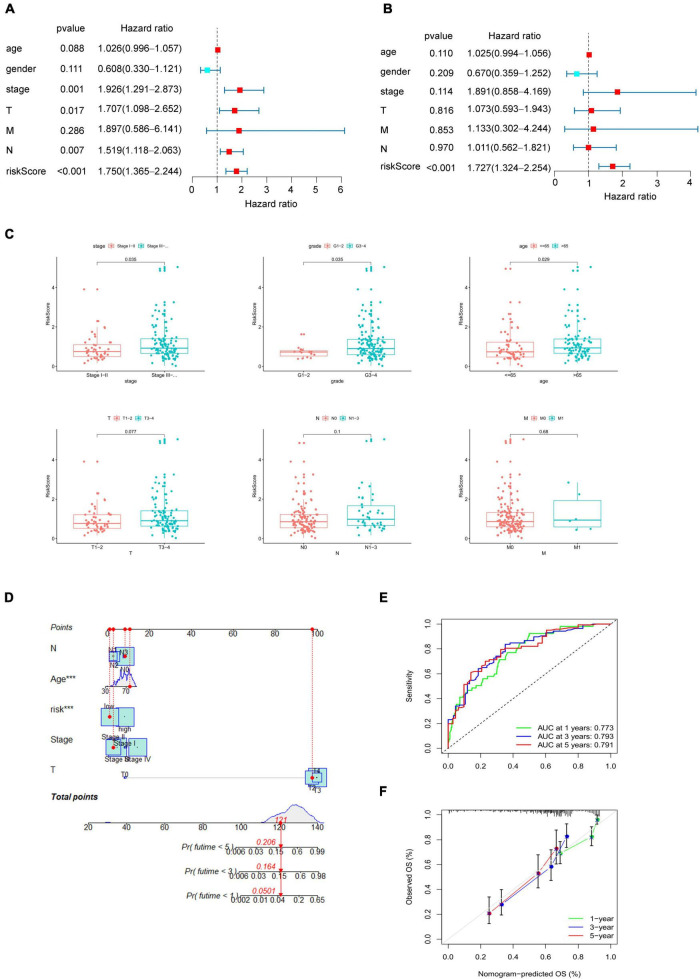
Clinical correlation of the present model. **(A)** Univariate Cox regression analysis to identify prognosis associated factors. **(B)** Multivariate Cox regression analysis to assess independent prognostic factors. The red and blue dots represent variables with hazard ratio > 1 and ≤ 1, respectively. **(C)** Relationship between the risk score and clinical pathological stage, histological grade, age, TNM status. **(D)** Nomogram of eRNAs-based systematic signature. *p* < 0.05 was considered statistically significant. **(E)** ROC curves of nomogram for 1–, 3– 5–years overall survival prediction. **(F)** Calibration diagram for the nomogram.

After proving the favorable ability of the present model to predict the survival of patients with bladder cancer, we next analyzed the capacity of the model as a prognostic factor compared with age, gender, pathological stage, TNM status. Univariable analysis was performed to examined factors may contribute to predict prognosis of bladder cancer. As shown in Figure 3A, pathological stage (HR = 1.926, *p* = 0.001), T status (HR = 1.707, *p* = 0.017), N status (HR = 1.519, *p* = 0.007), and risk score (HR = 1.750, *p* < 0.001), which play a role as risk factors in the prognosis of bladder cancer. Furthermore, multivariate analysis demonstrated that the risk score (HR = 1.727, *p* < 0.001, Figure 3B) was not only the independent risk factor for prognosis of bladder cancer, but the only risk factors compared with the commonly used clinicopathological variables (i.e., age, gender, pathological stage, and TNM status), which were not sufficient to serve as independent prognostic predictors (*p* > 0.05, Figure 3B).

To verify the reliability of the present model, we then analyzed the correlation between risk score and clinical variables, including age, gender, pathological stage, histological grade and TNM status. As Figure 3C shown, the increase of risk score indicated a higher pathological grade and histological stage, and the older may get higher risk score (*p* < 0.05, Figure 3C), which may suggest why the higher risk score, the worse prognosis. Finally, a systematic signature with promoted prediction accuracy was constructed, and a nomogram to predict 1-, 3-, and 5-year overall survival was prepared accordingly based on risk score and clinicopathological variables associated with the score including age, pathological stage, TN status (Figure 3D). The ROC curves demonstrated that the AUC for nomogram to predict the prognosis of bladder cancer patients in the first, third, and fifth year were 0.773, 0.793, and 0.791 respectively (Figure 3E), and the also shown a good accuracy by calibration diagram (Figure 3F).

### High score based on the risk model indicates promoting tumorigenesis and immunosuppression in tumor microenvironment

**FIGURE 4:**
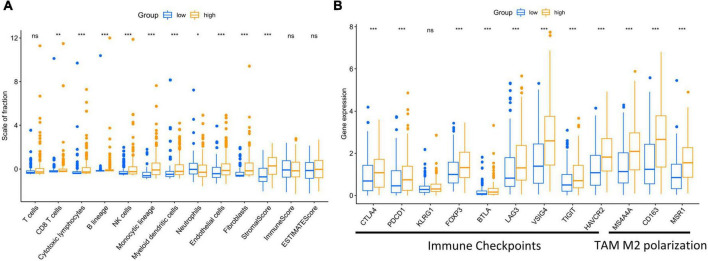
High risk score affected the infiltration of stromal and immune cells in tumor microenvironment, aggravating immunosuppression. **(A)** Different infiltration of stromal and immune cells was observed regarding risk score of the present model. **(B)** Immune checkpoint markers and type M2 tumor-associated macrophages markers were exhibited regarding risk score of the present model. *p* < 0.05 was considered statistically significant. symbol *, **, ***, ns mean *P*-value < 0.05, 0.01, 0.001, and no significance respectively.

To further explore the potential explanation for the correlation between higher risk score and unfavorable prognosis at cellular and molecular levels. We quantified and analyzed tumor-infiltrating immune cells and stromal cells, the main components of TME, by MCP-COUNTER method. The results shown that patients with higher risk score had greater infiltration of angiogenesis and cancer-associated fibroblasts (CAFs), as well as increased infiltration of CD8 + T cells, cytotoxic T cells and mononuclear macrophages (Figure 4A). Subsequently, we compared the expression levels of immune checkpoints and type M2 tumor-associated macrophage markers with high and low scores, and found that infiltrating T cells of patients with higher risk scores were generally immunosuppressive and unable to performed their immune functions (Figure 4B), despite the increased degree of immune cell infiltration, which demonstrated an inhibitory TME in patients with high score.

### Identification of eRNAs-related target genes and clustering analysis by weighted gene co-expression network analysis

To explore the functions eRNAs played in bladder cancer. We retrieved 26 target genes based on the eRic website. Then, 9 target genes that were differentially expressed between bladder cancer and normal samples were screened (*FDR* < 0.05, Supplementary Figure 3), in which upregulated expression of RHBG, PAQR6, CLK2, KRTCAP2, FLAD1, HCN3 were correlated with bad survival of patients with bladder cancer (Supplementary Figure 4). We next analyzed genes that were co-expressed with the particular 9 genes by a Pearson correlation coefficient test. As a result, 225 genes met the filter criteria (| correlation coefficient| > 0.5 and *p* < 0.001, Supplementary Table 1).

**FIGURE 5:**
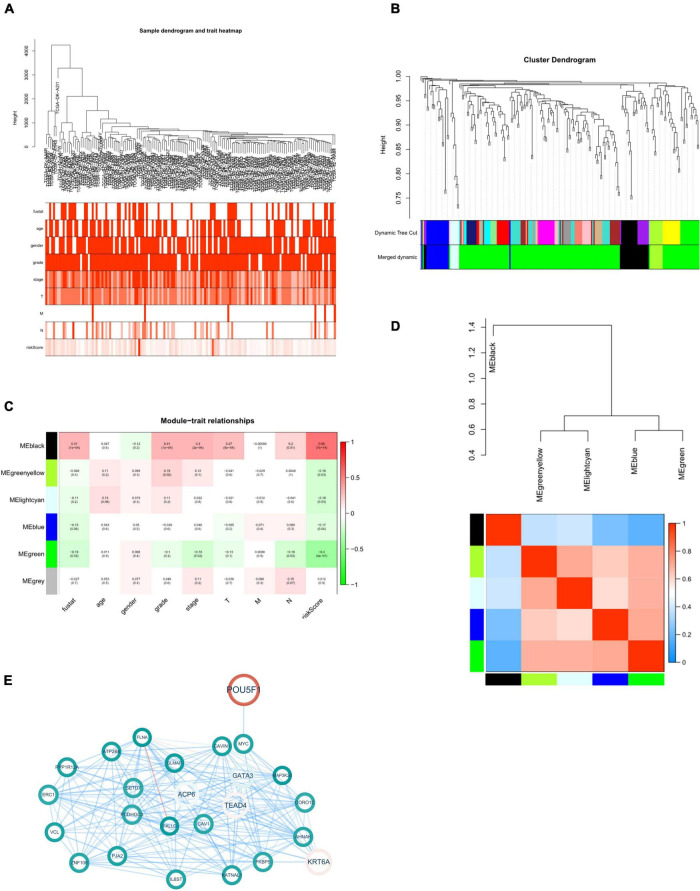
WGCNA of eRNAs-related genes. **(A)** Clustering dendrogram with clinical traits heatmap. **(B)** Identification of co-expressed modules. **(C)** Module-trait heatmap. **(D)** Interaction between co-expressed modules. The blue to red spectrum represents an increase in relevance. **(E)** Black module gene-based regulatory network. The red and blue lines represent positive and negative correlation, respectively. Color transparency and size of node were defined with gene expression level. *p* < 0.05 was considered statistically significant.

To further examine the role of 8 eRNAs from the present model in tumor progression, we next analyzed these 225 genes with a WGCNA algorithm to identify where eRNAs in the risk model might be involved in tumor progression. We constructed a dendrogram of clinical traits with hierarchical clustering (Figures 5A,B). Genes with similar expression patterns were classified into five distinct gene co-expressed modules. We next selected four of the five modules which was correlated with the clinicopathological variables, and constructed a protein-protein interaction network for the four modules to present the expressed relationship among these genes (Supplementary Figure 5).

Since the “black” one demonstrated a significant correlation with survival state, grade, stage and tumor infiltration of patients with bladder cancer (Figures 5C,D). Next, we constructed protein-protein interaction (PPI) network of 25 genes from this module to further explore the role of this module associated with risk eRNAs in tumor progression (Figure 5E).

### Functional enrichment analysis of eRNA-related target genes

**FIGURE 6:**
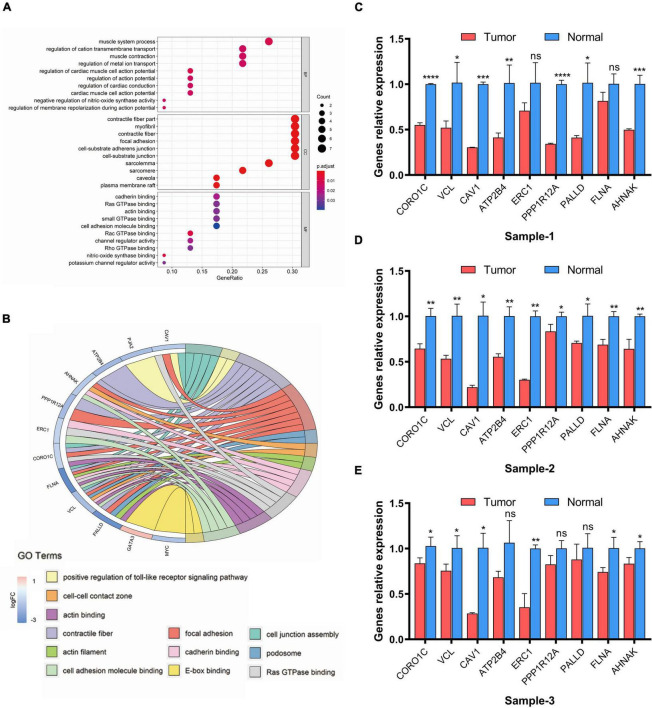
GO enrichment analysis of eRNAs-related genes. **(A)** Dot plot of enriched GO terms. **(B)** Chord plot of enriched GO terms. FDR < 0.05 was considered as statistically significant. **(C–E)** Real-time PCR analysis of related genes. Data are shown as the mean ± SEM. symbol *, **, ***, ns mean *P*-value < 0.05, 0.01, 0.001, and no significance respectively.

Next, GO enrichment analysis was performed to examine which functions these 25 eRNAs-related target genes significantly associated with clinical variables would have an impact on, resulting in tumorigenesis and progression in bladder cancer. The results suggested that these genes were mainly enriched in 41 biological processes, 12 molecular functions, and 21 cellular components (*FDR* < 0.05, Figure 6A). Remarkably, we found that these genes mainly enriched in the functions of mediating cell-extracellular matrix (ECM) and cell-cell adhesion, including focal adhesion, cell-substrate adherens junction, cell-substrate junction (*FDR* < 0.01), cadherin binding, cell adhesion molecule binding, Ras GTPase binding and actin binding (*FDR* < 0.05). To further demonstrate the function enrichment of this eRNAs-related regulatory network in cell-ECM and cell-cell adhesion, the chord chart was performed to display the related functions of GO set in a more complete way. As Figure 6B shown, the result demonstrated 12 functions associated with cell adhesion and invasion (*FDR* < 0.05), in which 9 genes mainly take part in regulation including CORO1C, VCL, CAV1, ATP2B4, ERC, PPP1R12A, PALLD, FLNA, AHNAK. These genes showing a down-regulated expression decrease the local adhesion of tumor cells and promote their invasion, which is correlated with the regulation of risk eRNAs.

Furthermore, to verified whether the expression levels of the 9 genes associated with regulation of eRNAs in the risk model change before and after the occurrence of tumor, we analyzed mRNA level of related genes between tumor and adjacent-free tissues from the clinic qRT-PCR (Figures 6C–E). The result suggested genes which participate in cell adhesion and invasion including CORO1C, VCL, CAV1, ATP2B4, ERC, PPP1R12A, PALLD, FLNA, AHNAK was confirmed down-regulated in tumor lesion compared with tissues adjacent to carcinoma, which was the same as our prediction in Figure 5E.

## Discussion

Bladder cancer is one of the primary causes of death worldwide, which has high rates of recurrence and progression, and most patients have a median overall survival of 15 months and a 5-year survival rate of 10% (26). The war between science and bladder cancer has not paused for these years, during which investigators have assessed new combinations of traditional treatments (surgery, chemotherapy, and radiotherapy) to prolong patient survival and improve their life quality (27, 28). Furthermore, immunotherapy has initiated a revolution in tumor therapeutic strategies (29, 30). Despite the advances in treatment, there is still an urgent need to develop effective biomarkers for early tumor diagnosis and follow-up monitoring (31).

The function of eRNAs in physiological activities or pathological change has been reported increasingly since their discovery in Tuan et al. (32). Indeed, eRNAs are emerging as potential diagnostic and therapeutic targets. Because of their function in leading transcription units in the early transcription of certain genes, eRNAs are regarded as possible signals to predict abnormal gene expression in the early stage of tumor development (33). Conversely, increasing evidence indicated that eRNAs directly take part in transcription initiation. Li et al. reported the functional importance of eRNAs in the actions of estrogen-regulated gene enhancers (34). Additionally, the transcription of p53 and other tumor-associated genes was enhanced by the production of eRNAs (35).

Study has proved the feasibility of long non-coding genes (LncRNA) as prognostic signature in bladder cancer (36). In this way, unstable nature of eRNAs after was produced makes an easier real time detection of their transcription level (15), which is feasible for us to construct an eRNA-based prognostic signature in bladder cancer and identify its clinical application. Therefore, we identified several risk eRNAs in The Cancer Genome Atlas dataset, and we constructed a prognostic model that may function as a favorable tool to predict bladder cancer prognosis. In fact, the present model precisely stratified bladder cancer patients into two subgroups with statistically different survival outcomes. Then, we recognized risk eRNAs could be regarded as independent risk factor to predict the prognosis of bladder cancer by univariate and multivariate Cox regression analysis. Remarkably, the commonly used clinicopathological variables (i.e., age, gender, pathological stage, and TNM status) were not sufficient to serve as independent prognostic predictors compared with the risk model. Additionally, we examined the correlation between the risk score and clinicopathological variables, and found that high score in the model is associated with the greater pathological stages and histological grade, and the results shown the positive correlation between the risk score and TNM status despite the results were not statistically significant which may require a larger sample size. We then constructed a systematic signature with a nomogram to facilitate clinical applicability of the risk model, which has a favorable prediction accuracy according to 0.773, 0.793, 0.791 of 1-, 3-, 5-AUCs for OS. The results above demonstrated a reliability of the risk model to predict the prognosis of patients with bladder cancer.

Tumor microenvironment (TME), a heterogenous collections of infiltrating and resident cells, cytokines and ECM, takes a critical role in tumor progression and metastasis (37). Here, we evaluated whether the risk eRNAs affect the major cellular components of TME including tumor infiltration immune cells (TIICs) and stromal cells in bladder cancer. We found that the higher risk score based on the risk model increased the infiltration of immune cells including CD8^+^ T cells, cytotoxic T cells and macrophages, and stromal cells like endothelial cells (ECs) and CAFs. Despite increasing immune cells infiltrated in the high score group, most of them was in inhibitory state, which resulted in a bad prognosis. Additionally, the increasing infiltration of ECs and CAFs always represents a bad prognosis (38).

After identified the relationship between the risk model and TME, we next try to explore the possible downstream mechanisms of eRNAs in tumor progression. First, we selected 9 of 26 genes from the target genes of the risk eRNAs, which were differentially expressed between bladder cancer and the normal. To further construct regulatory network and recognize the regulatory mechanisms, the correlation genes of the 9 target genes were selected (correlation coefficient > 0.5, *FDR* < 0.001). Next, we performed a co-expressed network and GO enrichment analysis to discover a possible mechanism of eRNA in tumor progression. After analysis with a WGCNA algorithm, we identified a prognostic-related co-expressed gene set (marked with black color). Survival state, histological grade, pathological stage, and TNM status of bladder cancer patients were all positively and precisely related to the “black” module gene expression. Furthermore, we found that genes in this module were enriched mainly in GO terms associated with tumor cell adhesion and invasion, including focal adhesion, cell-substrate adhesion junction, cadherin binding, and cell adhesion molecule binding, E-box binding (39–42). Meanwhile, genes participated in the regulation of these functions were selected, including CORO1C, VCL, CAV1, ATP2B4, ERC, PPP1R12A, PALLD, FLNA, AHNAK, which shown a down-regulated expression in bladder cancer. Then, qRT-PCR was performed to validate mRNA level of the nine genes enriched in cellular adhesion and invasion functions between tumor and para-cancerous tissues, which confirmed the regulation of eRNAs to target genes.

Although we have investigated the value of eRNAs in bladder cancer as comprehensively as possible with bioinformatics methods, this study did have some limitations. First, since it is hard to accurately recognize the location of genes encoding eRNAs, increasing the difficulty to determine their corresponding functions and regulation mechanisms. Second, further study is needed to confirm the roles related eRNAs takes *in vivo* and *in vitro*.

In summary, we constructed and verified an eRNA-based prognostic model that could be used as an independent prognostic signature to monitor bladder cancer progression and predict prognosis. In addition, we identified and analyzed a prognostic-related co-expressed gene set, which may further verify the reliability of eRNAs in bladder cancer prediction and identification of diagnostic and therapeutic targets, providing a new idea in tumor prognosis and progression monitor.

## Data availability statement

Publicly available datasets were analyzed in this study. This data can be found here: https://portal.gdc.cancer.gov/.

## Ethics statement

The studies involving human participants were reviewed and approved by the Ethics Committee of The First Affiliated Hospital of Air Force Military Medical University. The patients/participants provided their written informed consent to participate in this study. The animal study was reviewed and approved by the Ethics Committee of The First Affiliated Hospital of Air Force Military Medical University.

## Author contributions

LY, KZ, and DH: conceptualization, design, and project administration. ZX and CX: data acquisition, data analysis, and writing original draft. QW and SM: methodology. YL, SL, and SP: data interpretation. JT and XZ: writing review and editing. All authors contributed to the article and approved the submitted version.

## Footnotes

1
https://portal.gdc.cancer.gov/

2
https://hanlab.uth.edu/eRic/m3

3
https://www.r-project.org/
