# Supplementary material for: An enhancer RNA-based risk model for prediction of bladder cancer prognosis
Source: Front Med (Lausanne). 2022 Sep 14;9:979542. doi: 10.3389/fmed.2022.979542 (PMC9515318; doi:10.3389/fmed.2022.979542)
Supplement: Supplementary file 1 [file Data_Sheet_1.pdf]

SUPPLEMENTARY MATERIALS

Figure S1

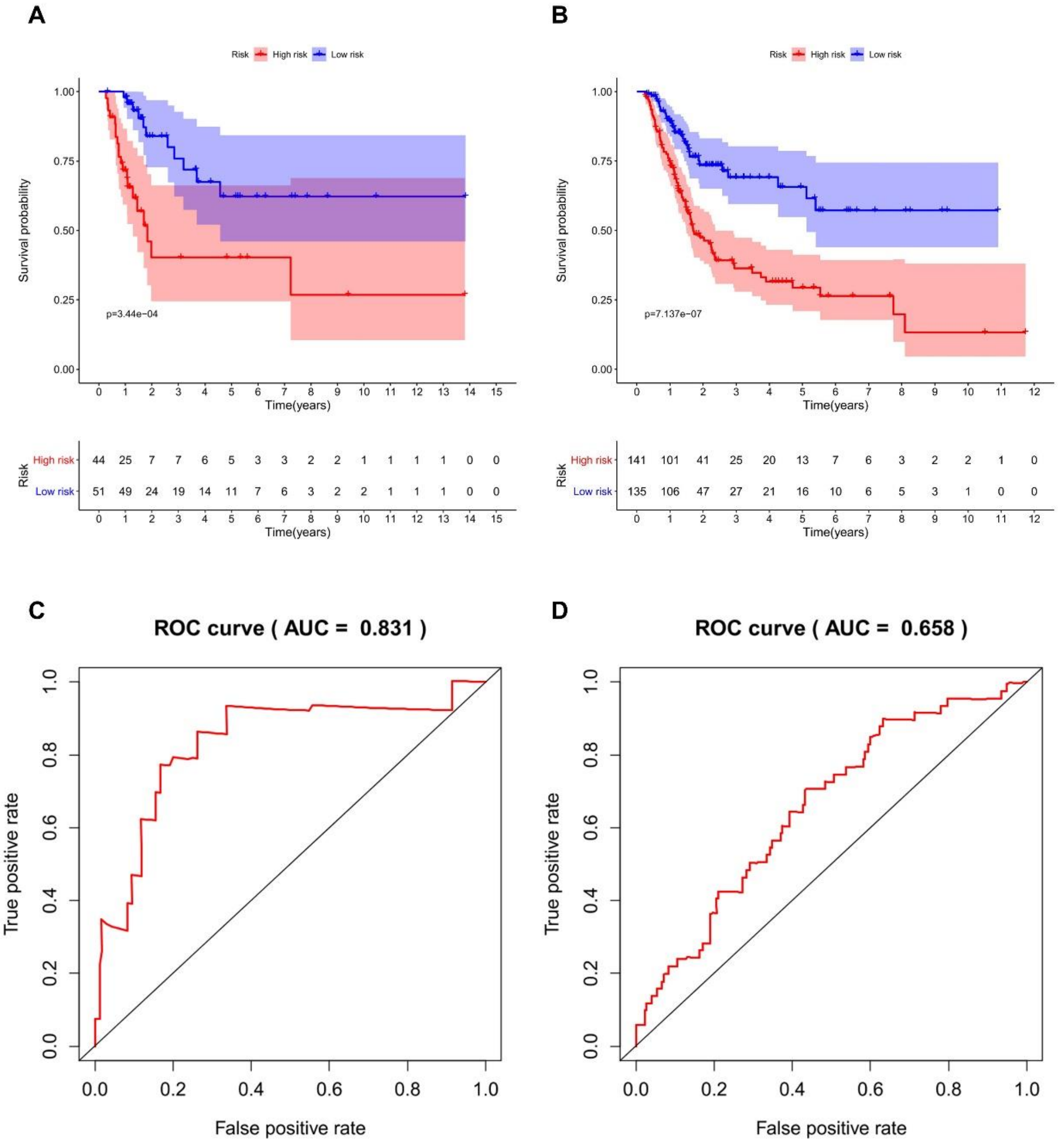

Figure S1. Validation sets to testify the accuracy of the risk model.
(A-B) Kaplan-Meier curve analysis for two of the validation. (C-D) 5-year ROC curves for the prediction accuracy of the risk model in validation sets. $p < 0.05$.

Figure S2

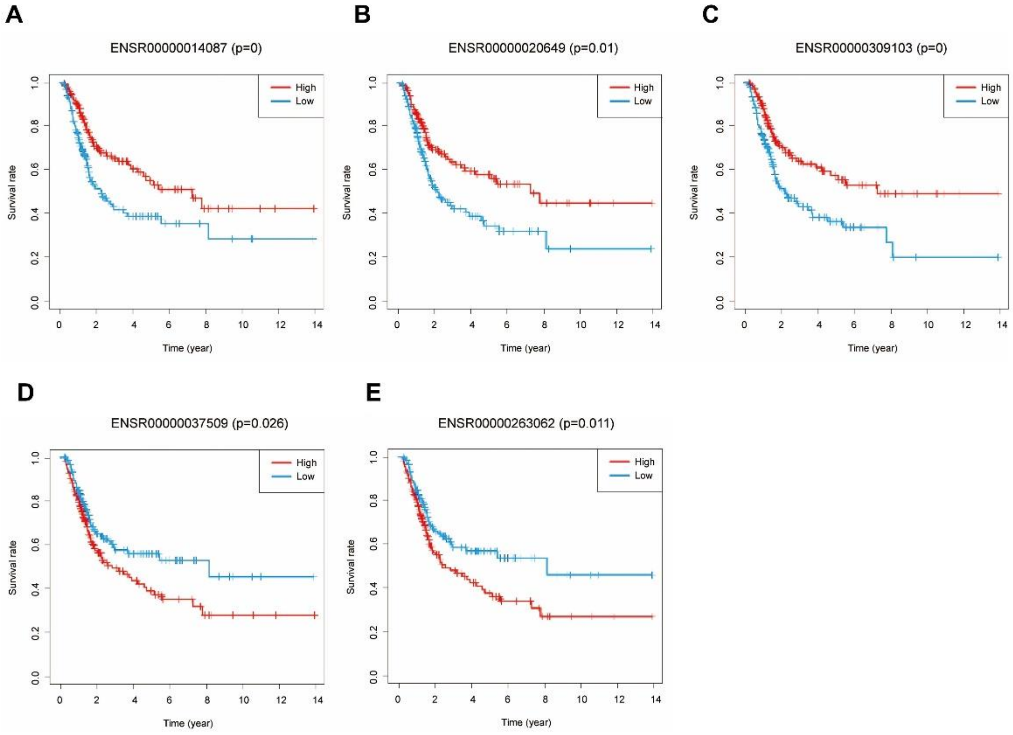

Figure S2. Relationship between survival and eRNAs.

(A) ENSR00000014087. (B) ENSR00000020649. (C) ENSR00000309103. (D) ENSR00000037509. (E) ENSR00000263062. The median expression level was used as cut-off value. $p < 0.05$ was considered statistically significant.

Figure S3

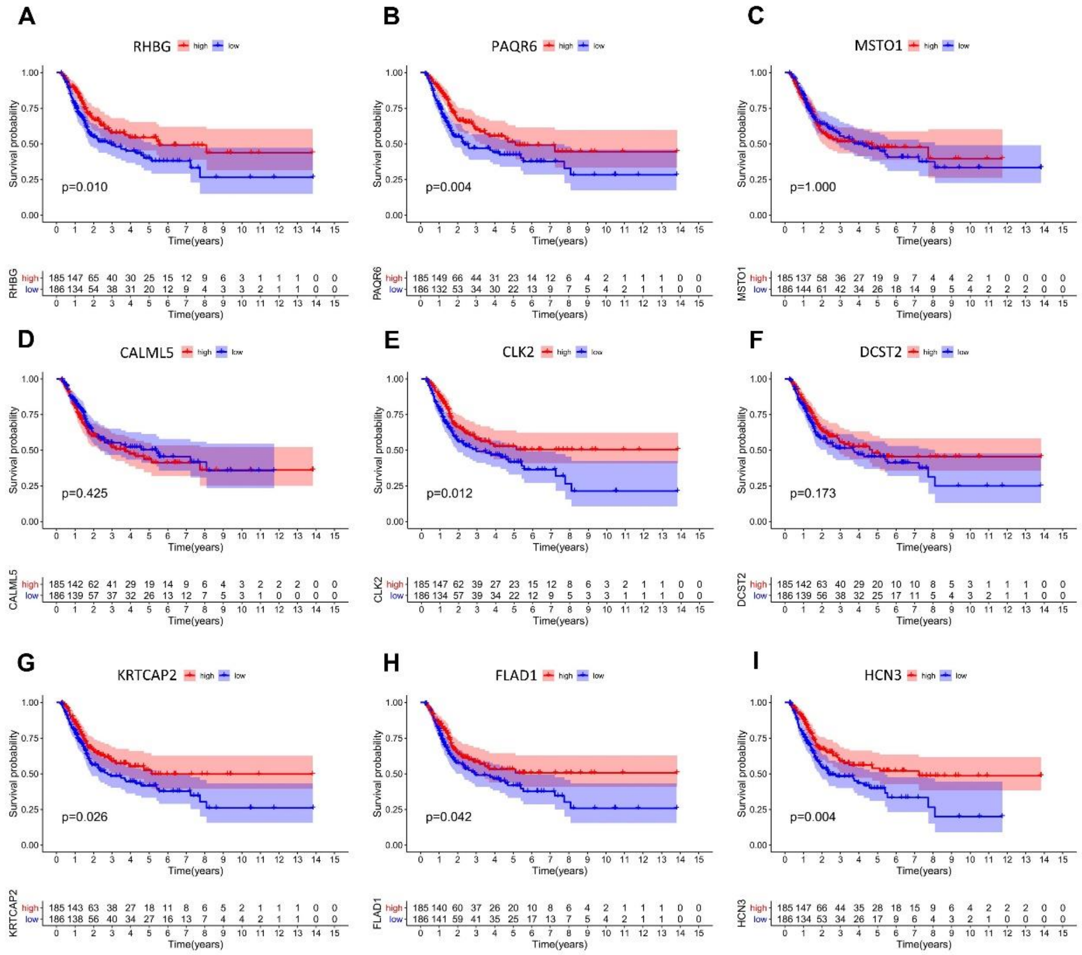

Figure S3. Survival analysis for the 9 target genes of the risk eRNAs.

(A-I) Kaplan-Meier curve analysis for related genes.

Figure S4

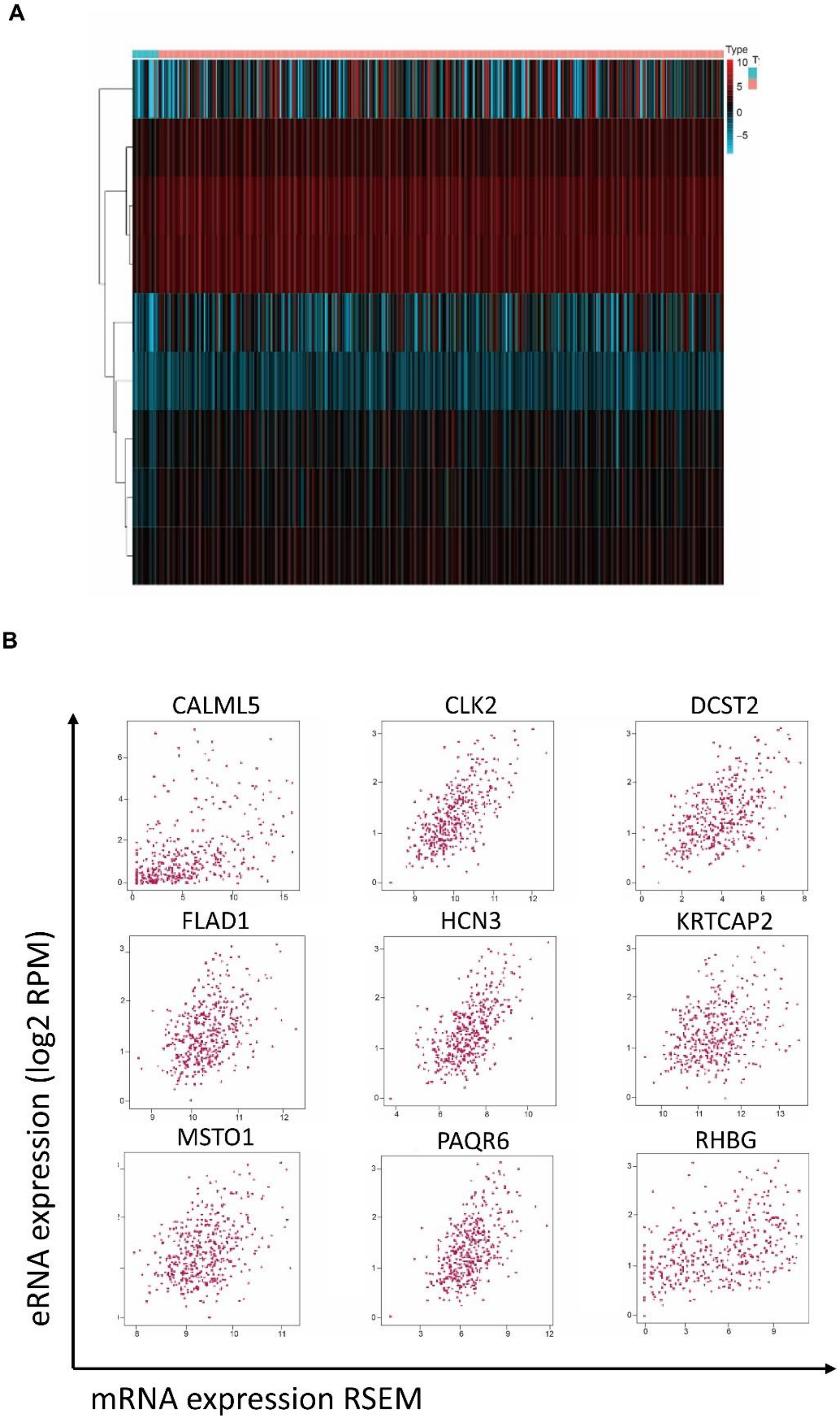

Figure S4. Identification of eRNAs-related target genes.

(A) Heatmap of eRNAs-related target genes. (B) Correlation between eRNAs and related target genes. FDR < 0.05 was considered as statistically significant.

Figure S5

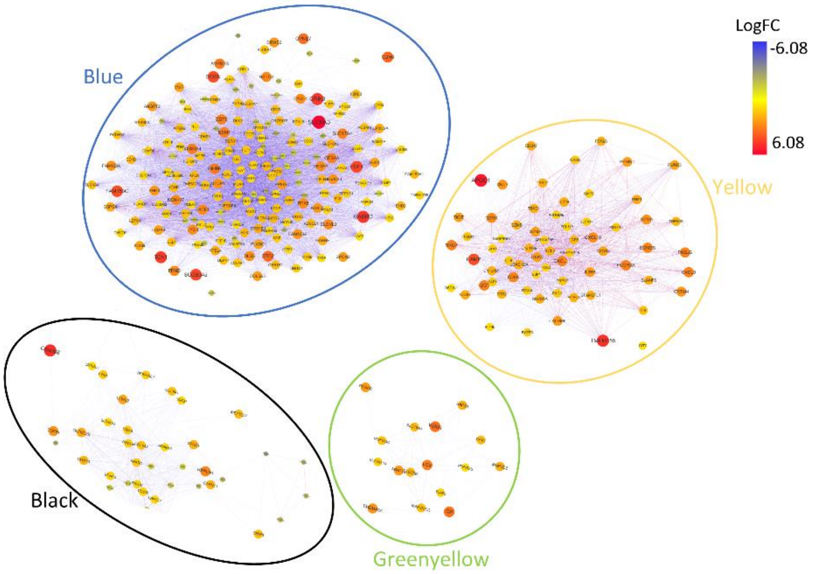

Figure S5. Protein-protein interaction network for the main modules of WGCNA analysis.
