# Supplementary material for: An enhancer RNA-based risk model for prediction of bladder cancer prognosis
Source: Front Med (Lausanne). 2022 Sep 14;9:979542. doi: 10.3389/fmed.2022.979542 (PMC9515318; doi:10.3389/fmed.2022.979542)
Supplement: Supplementary file 2 [file Table_1.docx]

| **Gene name** | **Forward primer (5’-3’)** | **Reverse primer (5’-3’)** |
| --- | --- | --- |
| AHNAK | AACTCAAGGGTCCAAAGTTCAAG | GAGAGACATCCACATCACCTTTC |
| ATP2B4 | ATGACGAACCCATCAGACCG | TCAGTGCATCCCTTGAACGC |
| CAV1 | GCGACCCTAAACACCTCAAC | ATGCCGTCAAAACTGTGTGTC |
| CORO1C | ATGAGGCGAGTGGTACGACA | ATCCCAGGTCACACGAGAAAC |
| ERC1 | GTCTGCCTTACGGTGTTCGG | GCTATGGTGTCACTAGCAACCC |
| FLNA | CTTATCGCGCTGTTGGAGGT | GCCACCGACACGTTCTCAA |
| PALLD | AAGAAGGCCAGTAGAACTGCT | AAGCGAAGTTTTCGTTCCAGG |
| PPP1R12A | TGAAGGCTGGATACCACTACA | TGCTCCTTGACCAATCAAAAACT |
| SLMAP | CCACGCTCTCGTCTGGTTT | CCTCGACTCAATCTCTGGCT |
| VCL | CTCGTCCGGGTTGGAAAAGAG | AGTAAGGGTCTGACTGAAGCAT |
|  |  |  |

**Supporting information. Primer sequence for Real-time quantitative PCR.**
